# HULC and H19 Played Different Roles in Overall and Disease-Free Survival from Hepatocellular Carcinoma after Curative Hepatectomy: A Preliminary Analysis from Gene Expression Omnibus

**DOI:** 10.1155/2015/191029

**Published:** 2015-06-07

**Authors:** Zongguo Yang, Yunfei Lu, Qingnian Xu, Bozong Tang, Cheol-Keun Park, Xiaorong Chen

**Affiliations:** ^1^Shanghai Public Health Clinical Center, Fudan University, Shanghai 201508, China; ^2^Samsung Medical Center, Sungkyunkwan University School of Medicine, Seoul 135-710, Republic of Korea

## Abstract

*Objective*. This study aimed to evaluate the relationships between long noncoding RNAs (lncRNAs) in tumor tissues and hepatocellular carcinoma (HCC) aggressiveness and survival. *Methods*. We correlated the lncRNAs in tumor tissues with HCC survival and clinicopathological features based on Gene Expression Omnibus expression profile GSE36376. *Results*. Eight lncRNAs and 240 HCC patients were included. Cox regression analysis indicated that HULC was a positive factor for HCC overall survival (HR = 0.885, 95% CI = 0.797–0.983, and *P* = 0.023) and disease-free survival time (HR = 0.913, 95% CI = 0.835–0.998, and *P* = 0.045). H19 and UCA1 were both demonstrated to be risk factors of HCC disease-free survival in multivariate Cox model (HR = 1.071, 95% CI = 1.01–1.137, and *P* = 0.022 and HR = 2.4, 95% CI = 1.092–5.273, and *P* = 0.029, resp.). But Kaplan-Meier method showed no significant association between UCA1 and HCC disease-free survival (log rank *P* = 0.616). Logistic regression demonstrated that H19 was overexpressed in HBV-infected patients (OR = 1.14, 95% CI = 1.008–1.29, and *P* = 0.037). HULC had a significant association with vascular invasion (OR = 0.648, 95% CI = 0.523–0.803, and *P* < 0.001). H19 and MEG3 were both considered to be risk factors for high AFP level (OR = 1.45, 95% CI = 1.277–1.646, and *P* < 0.001 and OR = 1.613, 95% CI = 1.1–2.365, and *P* = 0.014, resp.). *Conclusions*. Contributing to decreased susceptibility to vascular invasion, upregulation of HULC in tumor tissues was positively associated with HCC survival. In contrast, H19 overexpression might be risk factor for HCC aggressiveness and poor outcomes.

## 1. Introduction

Globally, HCC is the most common primary liver cancer, the fifth most common cancer, and the third most common cause of cancer-related deaths, representing around 5% of all cancers [1]. The occurrence and metastasis of HCC is a multistep process including tumor clinicopathological features, changes in signal transduction pathways, environmental makeup, gene mutations, and gene regulations [2, 3]. Recently, the widespread search for effective biomarkers of HCC is hoped to lead to earlier diagnosis and improve prognosis by allowing earlier intervention [3]. However, the molecular mechanism of HCC, especially gene regulatory mechanism, has not yet been fully elucidated.

It is well known that more than 90% of human genome undergoes transcription but does not code for proteins. lncRNAs are a class of noncoding RNA transcripts longer than 200 nucleotides with little or no protein-coding capacity [4]. As one of the key members of gene regulatory networks, lncRNAs take part in epigenetic regulation and are involved in diverse biological processes as well as disease pathogenesis [5, 6]. It is proved that lncRNAs can be used as potential carcinogenic and anticarcinogenic RNA [7]. In recent years, several reports revealed that lncRNAs were dysregulated in cancers [8–10], though their specific role in cancer proliferation, development, and progression was largely unclear. Increasing evidence has indicated that lncRNAs play a critical role in tumor biology, including tumor initiation, progression, and metastasis [11–13]. In addition, recent studies indicated that a number of lncRNAs are dysregulated in HCC, while their aberrant expressions are associated with tumorigenesis, metastasis, and diagnosis. Unfortunately, few data focused on the association of lncRNAs with prognosis of HCC [14, 15]. On the other hand, the roles of lncRNAs in HCC aggressiveness were controversial [10, 14].

Considering that increased evidence relates changes in expression levels of lncRNAs to cancers and controversial conflict existed before and few studies have evaluated the role of lncRNAs in HCC patients, further analysis to clarify this relationship between lncRNAs and HCC prognosis is urgently needed. This study set out to define the relationships between lncRNAs and survival and clinicopathological features in HCC patients, in the hope that the data may provide novel biomarker candidates as well as useful insights into the pathogenesis and progression of HCC.

## 2. Methods

### 2.1. Patients

240 tumor tissues containing no necrosis or hemorrhage were available from primary HCC patients who were treated with surgical resection or liver transplantation at Samsung Medical Center, Seoul, Korea, from July 2000 to May 2006. None of the patients received preoperative chemotherapy. Informed consent was obtained from each patient included in the study, and this study was approved by the institutional review board of Samsung Medical Center, Seoul, Korea, which is consistent with reports by Lim et al. [16]. The second analysis protocol was approved by the Ethics Committee of Shanghai Public Health Clinical Center, Fudan University.

### 2.2. Source of Data

Tumor tissues of HCC patients after curative hepatectomy in this study were profiled using Illumina HumanHT-12 V4.0 expression beadchip (Illumina Inc., San Diego, CA). The expression data was retrieved from Gene Expression Omnibus (GSE36376, http://www.ncbi.nlm.nih.gov/geo/) [16]. We restricted our search to genes within the lncRNAs, and eight lncRNAs previously reported in chronic liver diseases were included in our analysis.

### 2.3. End Points

The overall survival was defined as time from surgery to the date of death or last follow-up. The disease-free survival was defined as time from surgery to the date of tumor recurrence or death. The censoring time was defined as the final documented date of no evidence of tumor recurrence by imaging. As presented by Lim et al. [16], clinicopathological features of HCC patients including vascular invasion, major portal vein invasion, intrahepatic metastasis, multicentric occurrence, and nontumor liver pathology were all considered. Patient serum *α*-fetoprotein levels were evaluated and three phase dynamic computed tomography scans were performed at least once every 3 months after surgery until December 31, 2010. When tumor recurrence was suspected, precise diagnostic imaging was performed by magnetic resonance imaging.

### 2.4. Statistical Analysis

Student's* t*-test was used to compare means for normally distributed continuous data; Mann-Whitney* U *test was used for nonnormally distributed continuous data. Factors associated with the outcomes and clinicopathological features were assessed by univariate analysis and multivariate analysis separately using Cox and logistic regression. Only covariates significantly associated with outcomes at univariate analysis (two-sided *P* < 0.10) were included in the multivariate model. Results were reported as hazard ratios (HR) or odd ratios (OR) with 95% confidence intervals (CI). The Kaplan-Meier method was used to compare overall survival between different groups, and the log rank test was used to estimate the difference in survival. ROC curve was performed to evaluate predictive values of potential factors for HCC survival. Statistical analyses were performed using PASW Statistics software version 18.0 from SPSS Inc. (Chicago, IL, USA). All statistical tests were two-tailed, and differences with *P* < 0.05 were considered statistically significant.

## 3. Results

### 3.1. Patient Characteristics

As shown in Table 1, of the 240 patients included, 82.9% (199/240) were men and 17.1% (41/240) women with a median age of 53 (45–61) years and a mean body mass index (BMI) 24.2 ± 2.8 kg/m^2^. 77.5% (186/240) of patients had evidence of hepatitis B virus (HBV) infection, 8.3% (20/240) had evidence of hepatitis C virus (HCV) infection, and 5.8% (14/240) had evidence of alcohol use and for 20 patients no information was available. The median tumor size was 3.7 (2.5–6.15) cm. 55.4% (133/240) of patients had evidence of vascular invasion, 3.8% (9/240) had evidence of major portal vein invasion, 22.9% (55/240) had evidence of intrahepatic metastasis, and 5.4% (13/240) had evidence of multicentric occurrence. 2.1% (5/240) had direct invasion of adjacent organ. 36.3% (87/240) of these patients had an* alpha*-fetoprotein level more than 200 ng/mL and 47.9% (115/240) had a history of cirrhosis, 24.2% (58/240) had chronic active hepatitis, and 15% (36/240) had chronic persistent hepatitis. Tumor staging including AJCC and BCLC is also described in Table 1.

### 3.2. LncRNAs Expression Levels

Eight lncRNAs including H19, HOTAIR, MEG3, MALAT1, HULC, UCA1, HOXA13, and KCNQ1OT1 were considered in this analysis. LncRNAs expression levels between tumor and nontumor tissue of HCC patients are shown in Table 2. HOTAIR, MALAT1, HULC, and UCA1 were similarly expressed between tumor tissues and nontumor tissues in HCC patients (*P* = 0.448, 0.138, 0.085, and 0.373, resp.). MEG3, HOXA13, and KCNQ1OT1 were all overexpressed in HCC tumor tissues (*P* = 0.001, *P* < 0.001, and *P* = 0.004, resp.). However, H19 was expressed relatively higher in nontumor tissues compared to those in tumor tissues of HCC patients (*P* < 0.001).

### 3.3. HULC Was Associated with HCC Overall Survival

All lncRNAs included in the analysis were summarized in Table 3. Univariate analysis showed that HULC was a factor associated with overall survival in HCC patients (*P* = 0.005). When all these lncRNAs were evaluated by a multivariate model using enter selection, HULC was indicated to be a positive factor for HCC overall survival (HR = 0.885, 95% CI = 0.797–0.983, and *P* = 0.023).

**Figure 1:**
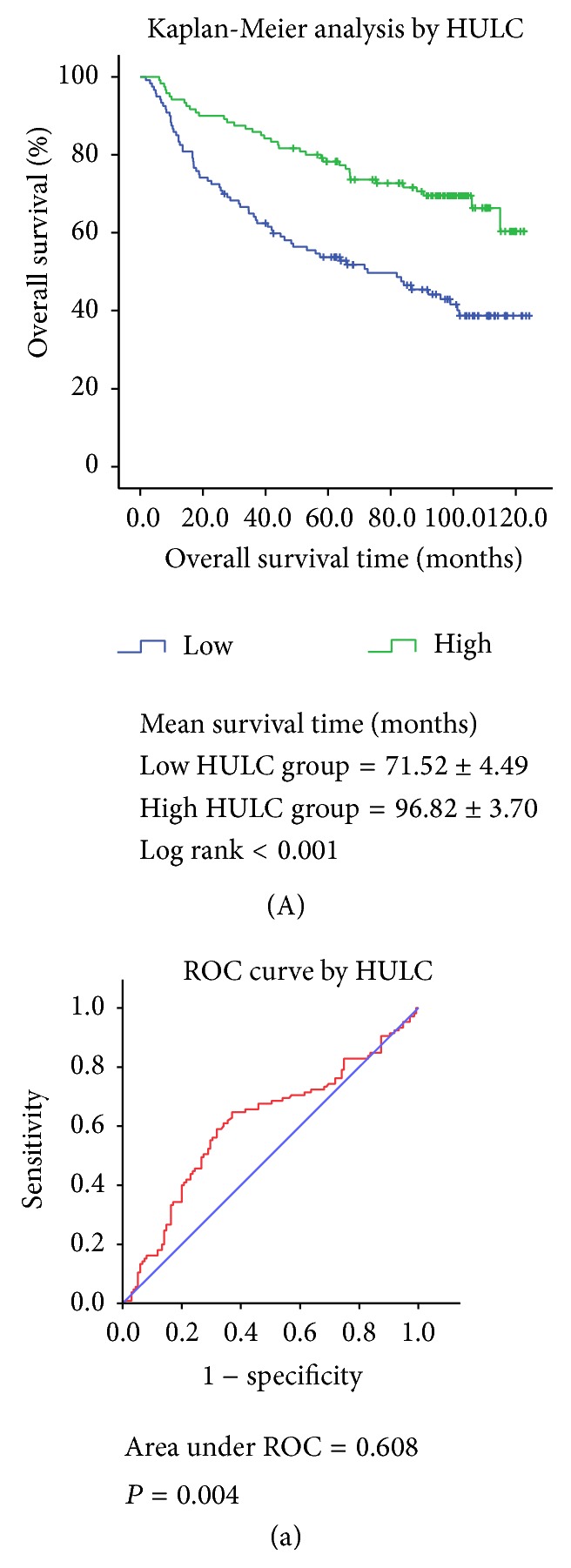
(A) Kaplan-Meier analysis of HCC overall survival by HULC with median cut-off; (a) ROC curve of HULC for predicting HCC overall survival.

We performed a Kaplan-Meier event analysis grouping by HULC identified to be significantly associated with survival presented above. For HULC, we grouped by median expression into a low expression and a high expression group with an 11.87 cut-off. As shown in Figure 1, this revealed that the higher the HULC expression, the greater the chance for longer survival (mean survival time, high = 96.82 ± 3.70 and low = 71.52 ± 4.49 months, resp.; log rank *P* < 0.001, Figure 1(A)). ROC curve also demonstrated that HULC expression level in tumor tissues could significantly predict HCC overall survival (area under ROC = 0.608; *P* = 0.004, Figure 1(a)).

### 3.4. H19, HULC, and UCA1 Were Associated with HCC Disease-Free Survival

Table 4 summarizes results from univariate and multivariate regression analyses of potential lncRNAs associated with HCC disease-free survival. H19, HULC, UCA1, and HOXA13 were all factors associated with disease-free survival in HCC patients (all *P* < 0.10). Furthermore, multivariate analysis using forward selection has shown that HULC should play a positive role in prolonging HCC disease-free survival time (HR = 0.913, 95% CI = 0.835–0.998, and *P* = 0.045). However, H19 and UCA1 were both demonstrated to be risk factors for HCC disease-free survival in multivariate Cox model (HR = 1.071, 95% CI = 1.01–1.137, and *P* = 0.022 and HR = 2.4, 95% CI = 1.092–5.273, and *P* = 0.029; resp.).

**Figure 2:**
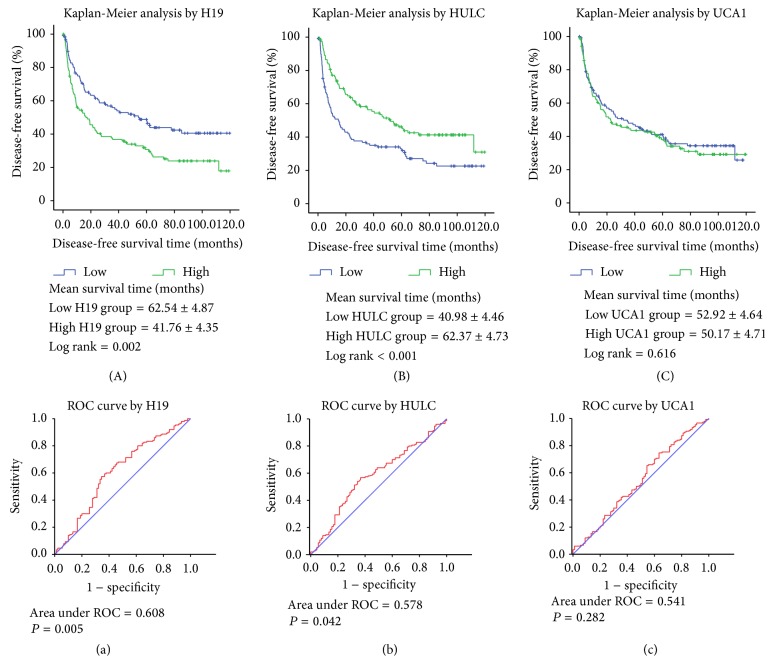
Kaplan-Meier analysis of HCC disease-free survival by H19 (A), HULC (B), and UCA1 (C); ROC curves of H19 (a), HULC (b), and UCA1 (c) for predicting HCC disease-free survival.

A Kaplan-Meier event analysis using log rank method was also performed further. For H19, HULC, and UCA1, we grouped by median expression into a low expression and a high expression group with cut-offs of 9.28, 11.87, and 6.51, respectively. As shown in Figure 2, this indicated that H19 overexpression in HCC tumor tissues might be a risk factor associated with HCC disease-free survival (mean survival time, high = 41.76 ± 4.35 and low = 62.54 ± 4.87 months, resp.; log rank *P* = 0.002, Figure 2(A)). In contrast, high HULC expression level is contributed to a better disease-free survival in HCC patients (mean survival time, high = 62.37 ± 4.73 and low = 40.98 ± 4.46 months, resp.; log rank *P* < 0.001, Figure 2(B)). Despite a significant correlation between UCA1 expression and HCC disease-free survival both in univariate and in multivariate regression analyses, the extent of the association by Kaplan-Meier event analysis with log rank method showed no significance (mean survival time, high = 50.17 ± 4.71 and low = 52.92 ± 4.64 months, resp.; log rank *P* = 0.616, Figure 2(C)). ROC curves revealed that H19 and HULC levels well predict HCC disease-free survival (area under ROC = 0.608, *P* = 0.005, and area under ROC = 0.578, *P* = 0.042, resp., Figures 2(a) and 2(b)), while no significance was found between UCA1 and HCC disease-free survival by ROC curve (area under ROC = 0.541, *P* = 0.282, Figure 2(c)).

### 3.5. Relationship between LncRNAs and HCC Clinicopathological Features

Only lncRNAs and clinicopathological features with significant association were shown in Table 5. Univariate logistic analysis showed that H19, MEG3, and MALAT1 expression were related with HBV infection (all *P* < 0.10). Multivariate model demonstrated that H19 and MALAT1 were overexpressed in HBV-infected patients (OR = 1.14, 95% CI = 1.008–1.29, and *P* = 0.037 and OR = 26.951, 95% CI = 2.022–359.284, and *P* = 0.013, resp.). Even though MEG3, HULC, and UCA1 were all associated with vascular invasion in univariate regression analysis (all *P* < 0.10), only HULC had a significant association with vascular invasion by multivariate model (OR = 0.648, 95% CI = 0.523–0.803, and *P* < 0.001). Moreover, lncRNAs including H19, MEG3, HULC, HOXA13, and KCNQ1OT1 were all factors associated with AFP level over 200 ng/mL (all *P* < 0.10), and when all these lncRNAs were evaluated by a multivariate model using forward selection, H19 and MEG3 were both considered to be risk factors for high AFP level (OR = 1.45, 95% CI = 1.277–1.646, and *P* < 0.001 and OR = 1.613, 95% CI = 1.1–2.365, and *P* = 0.014, resp.).

## 4. Discussion

Up to date, over 3,000 lncRNAs have been identified and only a small number of functional lncRNAs have been well characterized. Previous studies showed that lncRNAs are likely to be involved in many diverse biological processes, including cell proliferation, differentiation, cell cycle, apoptosis and invasion, marker of cell fate, and parental imprinting, indicating that they may play a major role in the regulation of eukaryotic genome [17–19]. Moreover, multiple lines of evidence link dysregulation of these lncRNAs to diverse human diseases, especially cancers [8–10]. Four known molecular functions of LncRNAs, including signal, decoy, guide, and scaffold, were summarized recently [14]: Firstly, lncRNAs can act as markers of functionally significant biological events by regulating transcriptional activity or pathway. Secondly, lncRNAs can bind and titrate away proteins or RNAs to indirectly exert biological functions in multiple kingdoms of life. Thirdly, the guide function of lncRNAs is that RNA binds specific protein(s) and then directs the localization of the resultant complex to specific targets. Fourthly, lncRNAs can serve as adaptors to bind relevant molecular components to regulate gene expression.

HULC (highly upregulated in liver cancer), 1.6 k nucleotide long, containing two exons but not translated, has been identified highly upregulated in HCC and colorectal cancer that metastasized to livers [20, 21]. The expression level of HULC is positively correlated with those of HBx in clinical HCC tissues. Additionally, HBx could upregulate HULC expression in human immortalized normal liver L-O2 cells and hepatoma HepG2 cells and upregulation of HULC by HBx could promote proliferation of hepatoma cells through suppressing p18 [22]. Based on the previous reports, HULC plays an important role in liver carcinogenesis and acts as an oncogenic ncRNA, but the role of HULC in predicting outcomes in HCC patients after curative therapy was largely unknown. In this study, we found that HULC was elevated in HCC tumor tissues compared with the corresponding nontumor tissues, even though there was no significance. Interestingly, HULC decreased HCC vascular invasion, which should be a positive factor for HCC prognosis. Furthermore, univariate and multivariate Cox regression analyses showed that upregulation of HULC in tumor tissues contributed to better outcomes both in overall survival and in disease-free survival. An interesting report by Liu et al. [10] elucidated that the variant genotypes of rs7763881 in HULC might contribute to decreased HCC susceptibility in HBV persistent carriers. Thus, full molecular mechanism of HULC in the natural history of HBV infection to HCC development should be investigated further.

The H19 gene encodes a 2.3 kb lncRNA that is exclusively expressed from the maternal allele, and it plays an important role in genomic imprinting during growth and development [14, 23]. Compared with healthy tissues, H19 is overexpressed in breast adenocarcinoma and is significantly associated with tumor values [24]. Recently, H19 was found to play an important role in HCC progression. In our analysis, H19 was overexpressed in HCC nontumor tissues. Moreover, correlated to chronic HBV infection and AFP evaluation, H19 overexpression was significantly associated with poor disease-free survival. That is, H19 might be a potential biomarker for HCC recurrence prediction. Previous study revealed that H19 was upregulated in HBV-associated HCC [14], which is inconsistent with our finding. Since hypoxia is a key trigger enhancing the expression of the H19 gene [25], the fact that more than half of HCC recurrence patients in this study were treated with transarterial chemoembolization (TACE, 86/240) and/or radiofrequency ablation (RFA, 38/240) might be an explanation for this.* In vitro*, a publication strongly suggested that H19 could act as a tumor suppressor [26], while other authors assumed that H19 acts as an oncogenic marker in humans [25, 27]. Although its role in tumorigenesis is debated, the prevailing view is that H19 behaves like an oncogene [28]. On the other side, AFP is the most widely tested biomarker in HCC. It is known that persistently elevated AFP levels can be used to help define at-risk populations and predict HCC recurrence [29], and H19 mRNA was coregulated with AFP in liver [30]. Considering previous reports and our results, we assumed that H19 should be a predictive marker for HCC recurrence.

UCA1, short for urothelial cancer associated 1, is believed to regulate the expression of several genes involved in tumorigenesis, embryonic development, or both [31]. It has been presented that UCA1 functions in regulation of embryonic development and in bladder cancer invasion and progression [32, 33], as well as breast tumor [34] and colorectal cancer [35]. Also, overexpression of UCA1 lncRNA could promote metastatic but not proliferation ability of tongue squamous cell carcinoma cells [36]. Our result showed that UCA1 is negatively associated with HCC disease-free survival. Considering no difference in UAC1 expression was found between tumor tissues and nontumor tissues in HCC patients, the predictive role of UAC1 in HCC disease-free survival should be evaluated further.

In most of human cancer cell lines, overexpression of MEG3 (maternally expressed gene 3) results in growth suppression, accumulation of p53 protein, and activation of p53 downstream targets [14]. In HCC tissues and cell lines, MEG3 expression is markedly reduced. It has been well revealed that the loss of MEG3 gene expression is associated with hypermethylation of the promoter region in HCC. Importantly, enforced expression of MEG3 in HCC cells significantly decreases both anchorage-dependent and anchorage-independent cell growth and induces cell apoptosis [37, 38]. However, our research showed that MEG3 elevated in HCC tumor tissues and correlated with AFP elevation in human. As a tumor suppressor gene, MEG3 is associated with tumorigenesis. An overall hypermethylation in specific MEG3 regions might result in permanent gene transcriptional silencing, consequent loss of its antiproliferative function contributing to oncogenesis [39].

MALAT1 (metastasis-associated lung adenocarcinoma transcript 1) was discovered as a prognostic marker for lung cancer metastasis but also has been linked to several other human tumor entities [40]. In our study, MALAT1 is related with chronic HBV infection, but not associated with HCC outcomes. Previously, MALAT1 was reportedly upregulated in HCC cell lines and clinical tissue samples. In addition, inhibition of MALAT1 in HepG2 cells could effectively reduce cell viability, motility, and invasiveness, while increasing the sensitivity to apoptosis [8, 41]. Therefore, MALAT1 may play an important role in tumor progression and could be a novel biomarker for predicting tumor prognosis [14].

Additionally, HOXA13 and KCNQ1OT1 were upregulated in HCC tumor tissues. A report by Quagliata et al. [42] revealed HOX genes deregulation to be involved in hepatocarcinogenesis, and HOXA13 are associated with HCC patients' clinical progression and predict disease outcome. Similarly, KCNQ1OT1 has been shown to be involved in multiple cancers. A short tandem repeat polymorphism within KCNQ1OT1 contributes to hepatocarcinogenesis, indicating that common genetic changes in KCNQ1OT1 may influence HCC risk [43]. Further functional studies are needed to validate these hypotheses and understand the roles of lncRNAs in HCC progress and prognosis.

In conclusion, contributing to decreased susceptibility to vascular invasion, upregulation of HULC in tumor tissues was positively associated with HCC survival. In contrast, H19, MEG3, and UCA1 might be risk factors for HCC aggressiveness and poor outcomes. Even though this study was based on data from a national data bank and no direct first-hand data were available and a lot of key questions about lncRNAs remain unsolved, exploration on lncRNAs field is shedding new light on our understanding of HCC. In patients with HBV-associated HCC, the expression of lncRNAs in HBV-HCC tissues was changed significantly compared with normal liver tissues, and lncRNAs played a pivotal role in the pathogenesis of HBV-HCC probably by mainly regulating the carcinoma-related signaling pathway and MAPK signaling pathway [44]. Unfortunately, lncRNA expression in healthy tissues was not available in this analysis. Further research focused on comparison between cancerous and normal samples and the potential action mode of lncRNAs must be conducted. In recent years, there is an exponential growth of studies on the biological functions of lncRNAs in human cancers, including HCC [14]. In the future, integration of lncRNA biology into HCC biology may further deepen our understanding of the mechanisms of HCC and provide novel applications for efficient, rapid, and specific diagnosis and effective treatments.

## Figures and Tables

**Table 1:** Baseline characteristics of HCC patients.

Characteristics	*n* = 240
Male, *n* (%)	199 (82.9)
Age, median (range), years	53 (45–61)
Body mass index, mean ± SD, kg/m^2^	24.2 ± 2.8
Etiology, HBV/HCV/alcohol/NA, *n*	186/20/14/20
Tumor size, median (range), cm	3.7 (2.5–6.15)
Vascular invasion, *n* (%)	133 (55.4)
Major portal vein invasion, *n* (%)	9 (3.8)
Intrahepatic metastasis, *n* (%)	55 (22.9)
Multicentric occurrence, *n* (%)	13 (5.4)
Direct invasion of adjacent organ, *n* (%)	5 (2.1)
AJCC stage, I/II/III/IV, *n*	102/100/33/5
BCLC stage, A/B/C, *n*	139/91/10
*α*-Fetoprotein >200 ng/mL, *n* (%)	87 (36.3)
Liver histology of nontumor tissues, *n* (%)	
Cirrhosis	115 (47.9)
Chronic active hepatitis	58 (24.2)
Chronic persistent hepatitis	36 (15.0)
Reactive hepatitis	11 (4.6)
Alcoholic hepatitis	11 (4.6)

NA: not available; HBV: hepatitis B virus; HCV: hepatitis C virus; AJCC: American Joint Committee on Cancer; BCLC: Barcelona Clinic Liver Cancer.

**Table 2:** LncRNA expression between tumor tissues and nontumor tissues of HCC patients.

LncRNAs	Tumor tissues	Nontumor tissues	*P* value
H19, median (range)	9.28 (6.10–14.54)	10.65 (7.58–13.83)	**<0.001**
HOTAIR, mean ± SD	6.19 ± 0.15	6.20 ± 0.14	0.448
MEG3, median (range)	5.98 (5.56–11.15)	5.92 (5.64–9.30)	**0.001**
MALAT1, mean ± SD	5.82 ± 0.13	5.84 ± 0.14	0.138
HULC, median (range)	11.87 (6.06–14.23)	11.77 (7.80–12.80)	0.085
UCA1, median (range)	6.51 (6.14–7.62)	6.51 (6.06–6.90)	0.373
HOXA13, median (range)	6.62 (6.11–8.57)	6.45 (5.92–6.81)	**<0.001**
KCNQ1OT1, median (range)	6.23 (5.45–10.21)	6.17 (5.63–6.63)	**0.004**

**Table 3:** Univariate and multivariate Cox regression analysis of lncRNAs associated with overall survival of HCC patients.

LncRNAs	Univariate analysis, HR (95% CI)	*P* value	Multivariate analysis, HR (95% CI)	*P* value
H19, per increase of 1 unit	1.025 (0.955–1.101)	0.488		
HOTAIR, per increase of 1 unit	3.059 (0.788–11.873)	0.106		
MEG3, per increase of 1 unit	1.079 (0.884–1.318)	0.455		
MALAT1, per increase of 1 unit	3.009 (0.673–13.45)	0.149		
HULC, per increase of 1 unit	0.859 (0.773–0.955)	0.005	0.885 (0.797–0.983)	**0.023**
UCA1, per increase of 1 unit	1.986 (0.842–4.685)	0.117		
HOXA13, per increase of 1 unit	0.715 (0.449–1.14)	0.159		
KCNQ1OT1, per increase of 1 unit	0.753 (0.415–1.366)	0.35		

HR: hazard ratios; CI: confidence intervals.

**Table 4:** Univariate and multivariate Cox regression analysis of lncRNAs associated with disease-free survival of HCC patients.

LncRNAs	Univariate analysis, HR (95% CI)	*P* value	Multivariate analysis, HR (95% CI)	*P* value
H19, per increase of 1 unit	1.068 (1.007–1.133)	0.028	1.071 (1.01–1.137)	**0.022**
HOTAIR, per increase of 1 unit	2.206 (0.711–6.842)	0.171		
MEG3, per increase of 1 unit	1.113 (0.947–1.307)	0.193		
MALAT1, per increase of 1 unit	2.765 (0.785–9.748)	0.114		
HULC, per increase of 1 unit	0.903 (0.825–0.989)	0.027	0.913 (0.835–0.998)	**0.045**
UCA1, per increase of 1 unit	2.352 (1.071–5.167)	0.033	2.4 (1.092–5.273)	**0.029**
HOXA13, per increase of 1 unit	0.697 (0.478–1.016)	0.06		
KCNQ1OT1, per increase of 1 unit	1.116 (0.712–1.75)	0.632		

HR: hazard ratios; CI: confidence intervals.

**Table 5:** Univariate and multivariate regression analysis of relationship between lncRNAs and HCC clinicopathological features.

LncRNAs	Univariate analysis, OR (95% CI)	*P* value	Multivariate analysis, OR (95% CI)	*P* value
HBV infection				
H19, per increase of 1 unit	1.143 (1.013–1.29)	0.03	1.14 (1.008–1.29)	**0.037**
MEG3, per increase of 1 unit	1.503 (0.949–2.383)	0.083		
MALAT1, per increase of 1 unit	28.164 (2.179–364.071)	0.011	26.951 (2.022–359.284)	**0.013**

Vascular invasion				
MEG3, per increase of 1 unit	1.425 (1.034–1.963)	0.031		
HULC, per increase of 1 unit	0.64 (0.522–0.786)	<0.001	0.648 (0.523–0.803)	**<0.001**
UCA1, per increase of 1 unit	2.952 (0.822–10.601)	0.097		

AFP over 200 ng/mL				
H19, per increase of 1 unit	1.526 (1.352–1.723)	<0.001	1.45 (1.277–1.646)	**<0.001**
MEG3, per increase of 1 unit	2.304 (1.584–3.351)	<0.001	1.613 (1.1–2.365)	**0.014**
HULC, per increase of 1 unit	0.855 (0.726–1.007)	0.06		
HOXA13, per increase of 1 unit	0.547 (0.288–1.039)	0.065		
KCNQ1OT1, per increase of 1 unit	2.557 (0.992–6.592)	0.052		

HBV: hepatitis B virus; AFP: *α*-fetoprotein; OR: odd ratios; CI: confidence intervals.
